# Ubiquitous Systems towards Green, Sustainable, and Secured Smart Environment

**DOI:** 10.1155/2015/281921

**Published:** 2015-03-19

**Authors:** Jong-Hyuk Park, Yi Pan, Han-Chieh Chao, Neil Y. Yen

**Affiliations:** ^1^Seoul National University of Science and Technology, 172 Gongreung 2-dong, Nowon-gu, Seoul 139-743, Republic of Korea; ^2^Georgia State University, 25 Park Place, Room 744, Atlanta, GA 30302-5060, USA; ^3^National Ilan University, No. 1, Section 1, Shen-Lung Road, YiLan 260, Taiwan; ^4^The University of Aizu, Tsuruga, Ikkimachi, Matsunaga, Aizuwakamatsu, Fukushima 965-8580, Japan

Expected goal of ubiquitous system is to prompt a tight integration among information, human beings, and their coexisting environment. In line with matured techniques, making systems lighter, smaller, but powerful becomes an emerging trend. To develop a smart environment, improvements to performance, sustainability, and security of conventional systems are essential. Taking smart home, for instance, all devices are connected to specific area networks and smart meters and are controlled through demand side management techniques, where they can make sure all the devices are functional and stay controlled. With real-time pricing schemes, these techniques can achieve efficient energy usage as well as monetary expense reduction and also enable the integration of renewable energy resources and facilitate the peak-to-average load ratio reduction for balancing energy consumption. Although advantages can be envisioned, the design of secured ubiquitous systems and making them right-to-the-needs are highly desired. And this special issue intends to tackle such deluge issues in ubiquitous systems design, prompting a green, sustainable, and secured computing environment.

During the past few months, we received over 80 submissions from at least 15 different countries where the corresponding authors were majorly counted by the deadline for manuscript submission. All these submissions were considered significant in the area of the promising applications in ubiquitous systems, but however, only three-fifth of them passed the first-round examination which is based on a strict and rigorous review policy. After a two-round review process, only 22 papers were accepted for being included in this issue. These accepted papers mainly look at our issue from the perspectives of ubiquitous sensing, routing, and optimization algorithm for wireless sensor network, data analysis in ubiquitous environment, ubiquitous service for e-learning and e-healthcare, privacy and security in smart environment, social technique in ubiquitous network design, location-based service and its fundamental technique adjustment, authentication in ubiquitous mobile cloud, and storage and management of large-scale data, which brought lively and focused discussions to the publics.

“Secure Cooperative Spectrum Sensing for the Cognitive Radio Network Using Nonuniform Reliability” addresses the importance of reliability in a cognitive radio network under a highly ubiquitous environment. A cooperative spectrum sensing approach where users are assigned nonuniform reliability is primarily considered. The nonuniform reliabilities serve as identification tags and are used to isolate users with malicious behavior. Three different strategies under this idea are presented in order to ignore unreliable and malicious users in the network. Considering only reliable users for global decision improves sensing time and decreases collisions in the control channel. The proposed schemes reduce the number of sensing reports and increase the inference accuracy.

“DS-ARP: A New Detection Scheme for ARP Spoofing Attacks Based on Routing Trace for Ubiquitous Environments” addresses the issue of attack detection in an open ubiquitous environment. A new detection scheme for ARP (Address Resolution Protocol) spoofing attacks using a routing trace, which can be used to protect the internal network, is proposed and implemented. Tracing routing can find the change of network movement path. The proposed scheme provides high constancy and compatibility because it does not alter the ARP protocol. In addition, it is simple and stable, as it does not use a complex algorithm or impose extra load on the computer system.

“Resource Management Scheme Based on Ubiquitous Data Analysis” introduces an idea concerning well management and analysis of ubiquitous data. An adaptive web process manager scheme based on the analysis of web log mining is proposed. The number of web processes is controlled through prediction of incoming requests, and accordingly, the web process management scheme consumes the least possible web transaction resources. Authors adopted real web trace data to demonstrate the improvement in performance of the proposed scheme through a series of experiments.

“An Efficient Algorithm for Maximizing Range Sum Queries in a Road Network” discusses an open issue in the road network. It addresses the problem of processing MaxRS (Maximizing Range Sum) queries in a road network. The external-memory algorithm suited for a large road network database is proposed. In addition, in contrast to the existing methods, which retrieve only one optimal location, our proposed algorithm retrieves all the possible optimal locations. The performance of the proposed algorithm is evaluated through the simulations and the real-world experiments.

“A Service Based Adaptive U-Learning System Using UX” introduces a promising application of ubiquitous systems in e-learning environment. It focuses on providing the learning material and processes of courses by learning units using the services in a ubiquitous computing environment. And it also investigates functions that support users' tailored materials according to their learning style. That is, the user's data and their characteristics are analyzed in accordance with their user experience. It subsequently applies the learning process to fit on their learning performance and preferences. Finally, how the proposed system outperforms learning effects to learners better than existing techniques is demonstrated and proved by the experiment results.

“An Evaluation and Implementation of Rule-Based Home Energy Management System Using the Rete Algorithm” discusses the use of smart sensors to prompt the development of home energy management system (HEMS). This paper evaluates a rule-based HEMS using the Rete algorithm by simulation. In the simulation results, the proposed system distributes the load for rule processing to nodes and has achieved reducing the maximum load of node compared with server-based method and Not-Rete method.

“Preserving Differential Privacy for Similarity Measurement in Smart Environments” proposed a secure protocol to compute coefficient function within differential privacy model for data privacy protection in smart environments. Unlike the existing solutions, our protocol can facilitate more than one request to compute coefficient function without modifying the protocol. Our solution ensures privacy protection for both the inputs and the computed coefficient function results. Although the target area is a smart environment, the same solution can be applied to other related areas such as pervasive or ubiquitous computing and intelligent environments.

“Applying Dynamic Priority Scheduling Scheme to Static Systems of Pinwheel Task Model in Power-Aware Scheduling” outlines that the dynamic priority scheduling results in power-aware scheduling could be applied to pinwheel task model. This method is more effective than adopting the previous static priority scheduling methods in saving energy consumption and, for the system being still static, it is more tractable and applicable to small sized embedded or ubiquitous computing. In addition, a novel power-aware scheduling algorithm which exploits all slacks under preemptive earliest-deadline first scheduling which is optimal in uniprocessor system is introduced. The dynamic priority method presented in this paper could be applied directly to static systems of pinwheel task model. The simulation results show that the proposed algorithm with the algorithmic complexity of *O*(*n*) reduces the energy consumption by 10–80% over the existing algorithms.

“Density-Based Penalty Parameter Optimization on C-SVM” discusses the density-based penalty parameter optimization in C-SVM (support vector machine) algorithm. In LIBSVM, C-SVM model is improved by the number proportion of the positive instances to the negative ones, but however, the spatial distribution of the initial instances has not been involved in the model training process. This paper aims to provide a better solution of the value of parameter; thus under the same conditions it can achieve a relatively accurate classification result.

“A Socially Aware Routing Based on Local Contact Information in Delay-Tolerant Networks” introduces an efficient routing scheme by using a node's local contact history and social network metrics. Each node first chooses a proper relay node based on the closeness to the destination node. A locally computed betweenness centrality is additionally utilized to enhance the routing efficiency. Through intensive simulation, we finally demonstrate that our algorithm performs efficiently compared to the existing epidemic or friendship routing scheme.

“An Effective Approach to Improving Low-Cost GPS Positioning Accuracy in Real-Time Navigation” presents an effective approach to improving the positioning accuracy of a low-cost GPS receiver for real-time navigation. The proposed method precisely estimates position by combining vehicle movement direction, velocity averaging, and distance between waypoints using coordinate data (latitude, longitude, time, and velocity) of the GPS receiver. The experimental results show that the proposed approach outperforms other state-of-the-art methods in terms of positioning accuracy compared to the other two state-of-the-art methods: recursive averaging and ARMA interpolation.

“Sloped Terrain Segmentation for Autonomous Drive Using Sparse 3D Point Cloud” proposes a framework for segmenting the ground in real time using a sparse three-dimensional (3D) point cloud acquired from undulating terrain. A sparse 3D point cloud can be acquired by scanning the geography using light detection and ranging (LiDAR) sensors. For efficient ground segmentation, 3D point clouds are quantized in units of volume pixels (voxels) and overlapping data is eliminated. The nonoverlapping voxels to two dimensions by implementing a lowermost heightmap are greatly reduced. The ground area is determined on the basis of the number of voxels in each voxel group. We execute ground segmentation in real time by proposing an approach to minimize the comparison between neighboring voxels.

“Two-Layer Fragile Watermarking Method Secured with Chaotic Map for Authentication of Digital Holy Quran” presents a novel watermarking method to facilitate the authentication and detection of the image forgery on the Quran images. Two layers of embedding scheme on wavelet and spatial domain are introduced to enhance the sensitivity of fragile watermarking and defend the attacks. Discrete wavelet transforms are applied to decompose the host image into wavelet prior to embedding the watermark in the wavelet domain. The watermarked wavelet coefficient is inverted back to spatial domain; then the least significant bits are utilized to hide another watermark. A chaotic map is utilized to blur the watermark to make it secure against the local attack. The proposed method allows high watermark payloads, while preserving good image quality.

“Study on Chaotic Fault Tolerant Synchronization Control Based on Adaptive Observer” considers the abrupt fault in chaotic system, employs an observer-based active fault tolerant approach, diagnoses fault online using adaptive observer, and achieves synchronization of chaotic system effectively. the effectiveness of our method is verified through numerical simulations; experimental results showed that synchronization can be achieved no matter whether the system failed or not. Finally, the proposed method is also applied to chaotic secure communications.

“Secure and Privacy Enhanced Gait Authentication on Smart Phone” proposes a novel gait based authentication using biometric cryptosystem to enhance the system security and user privacy on the smart phone. Extracted gait features are merely used to biometrically encrypt a cryptographic key which is acted as the authentication factor. Gait signals are acquired by using an inertial sensor named accelerometer in the mobile device and error correcting codes are adopted to deal with the natural variation of gait measurements. The proposed system on a dataset consisting of gait samples of 34 volunteers is evaluated. The results achieve the lowest false acceptance rate (FAR) and false rejection rate (FRR) of 3.92% and 11.76%, respectively, in terms of key length of 50 bits.

“A Secure and Efficient Audit Mechanism for Dynamic Shared Data in Cloud Storage” proposes a secure and efficient audit mechanism for dynamic shared data in cloud storage. The proposed scheme prevents a malicious cloud service provider from deceiving an auditor. Moreover, it devises a new index table management method and reduces the auditing cost by employing less complex operations. It is proved to have the resistance against some attacks and show less computation cost and shorter time for auditing when compared with conventional approaches. The results present that the proposed scheme is secure and efficient for cloud storage services managing dynamic shared data.

“Controlled Bidirectional Quantum Secure Direct Communication” proposes a controlled bidirectional quantum secure direct communication using a nonlocal swap gate to simultaneously exchange quantum information or classical messages without transmitting the qubits carrying the secret messages. In addition, the proposed protocol uses minimal quantum resources for legitimate users to transmit any unknown qubits in controlled bidirectional QSDC protocols. It is secure against eavesdropping attacks, and the controller has no access to the quantum information or secret messages in the protocol.

“Analyzing the Impact of Storage Shortage on Data Availability in Decentralized Online Social Networks” proposes a data availability model over storage capacity for DOSNs. Further, a novel method is proposed to predict the data availability on the fly. Extensive simulation experiments have been conducted. The results show that the proposed data availability method is able to capture the relation between data availability and storage capacity effectively and that the on-the-fly prediction method can predict the level of data availability accurately.

“Compressed Sensing Based Fingerprint Identification for Wireless Transmitters” proposes a fingerprint identification method for wireless transmitter signal based on compressed sensing. Complex analytical wavelet transform is used to obtain the envelope of the transient signal, and features are extracted from the envelope using the compressed sensing theory. A feature selection utilizing minimum redundancy maximum relevance (mRMR) is employed to obtain optimal feature subsets for identification. Finally, the recognition of 8 wireless transmitters by the SVM recognizer and BP neural network recognizer is completely performed.

“User Localization in Complex Environments by Multimodal Combination of GPS, WiFi, RFID, and Pedometer Technologies” proposes a conceptual design of a general localization platform using combination of multiple localization technologies. The combination is realized by dividing spaces into grid points. To demonstrate this platform, a system with GPS, RFID, WiFi, and pedometer technologies is established. Experiment results show that the accuracy and availability are improved in comparison with each technology individually.

The era of ubiquitous computing and its extended systems, applications, and services has drawn attentions to the publics and indeed caused great changes to our daily lives. With the success in the organization of this special issue in The Scientific World Journal, it becomes possible for researchers (and interesting readers as well) who have been engaged in this or related areas to receive state-of-the-art information, gain experiences, and further bring about the benefits in this promising area of study. We, the guest editors, also envision the advanced stimulation of development of innovative services and solutions in this area can be achieved in the coming future.

***Jong-Hyuk Park*** ***Jong-Hyuk Park*** *Yi Pan* *Yi Pan* *Han-Chieh Chao* *Han-Chieh Chao* ***Neil Y. Yen*** ***Neil Y. Yen***

